# Clinical characteristics and longitudinal chest CT features of healthcare workers hospitalized with coronavirus disease 2019 (COVID-19)

**DOI:** 10.7150/ijms.48696

**Published:** 2020-09-21

**Authors:** Huaping Liu, Shiyong Luo, Hailan Li, Youming Zhang, Chiyao Huang, Xili Li, Yiqing Tan, Mingna Chen

**Affiliations:** 1 Department of Radiology, The Third Xiangya Hospital, Central South University, Changsha 410013, Hunan Province, China.; 2 Department of Radiology, Wuhan Third Hospital (Tongren Hospital of Wuhan University); Wuhan 430060, Hubei Province, China.; 3 Department of Radiology, Hunan Provincial People's Hospital (The first affiliate hospital of Hunan normal university), Changsha 410000, Hunan Province, China.; 4 Department of Radiology, Xiangya Hospital, Central South University, Changsha 410008, Hunan Province, China.; 5 Department of Chinese Medicine, First Clinical College of China Three Gorges University; Yichang 443000, Hubei Province, China.; 6 Department of Ultrasonography, Xiangya Hospital, Central South University, Changsha 410008, Hunan Province, China.

**Keywords:** COVID-19, SARS-CoV-2, CT, healthcare workers

## Abstract

**Rationale:** The clinical data and corresponding dynamic CT findings were investigated in detail to describe the clinical and imaging profiles of COVID-19 pneumonia disease progression.

**Methods:** Forty HCWs with COVID-19 were included in this study and 30 enrolled for imaging assessment. Disease was divided into four stages based on time from onset: stage 1 (1-6 days), stage 2 (7-13 days), stage 3 (14-22 days), and stage 4 (> 22 days). Clinical wand imaging data were analyzed retrospectively.

**Results:** The cohort included 33 female and 7 male cases, with a median age of 40 years. Six had underlying comorbidities. More than half of the cases were nurses (22, 55%). Each stage included 39, 37, 34 and 32 CTs, respectively. Bilateral lesions, multifocal lesions and lesions with GGO pattern occurred in both lower lobes at all stages. The crazy-paving pattern (20, 54%), air bronchogram (13, 35%), and pleural effusion (2, 5%) were the most common CT features in stage 2. Consolidation score peaked in stage 2 whereas total lesions score peaked in stage 3.

**Conclusions:** COVID-19 pneumonia in HCWs has a potential predilection for younger female workers. Stage 2 of COVID-19 pneumonia may be the key period for controlling progression of the disease, and consolidation scores may be an objective reflection of the severity of lung involvement.

## Introduction

As of August 22, 2020, the cumulative confirmed cases of COVID-19 had exceeded 22,000,000 globally, with the death toll >790,000 1. America had become the world's COVID-19 epicenter with an overwhelming quantity of confirmed cases (>12,160,000) 1. The epidemics in the Europe, South-East Asia and Eastern Mediterranean were also not optimistic 1. Under these circumstances, millions of people have chosen to stay at home to reduce the risk of infection. Conversely, healthcare workers (HCWs) worldwide must work to help contain the disease outbreak and tend to those who have fallen ill. Epidemiological data show that HCWs are at an increased risk of exposure. On February 17, 2020, the Chinese Center for Disease Control and Prevention (CCDCP) reported that 3,019 HCWs in China had been infected with SARS-CoV-2, the virus that causes COVID-19 2. In Italy, 20% of HCWs have become infected 3, and by February 20 in Spain, more than 9,400 medical professionals were infected 4. HCWs have suffered from physical and mental disorders, and at times even death due to COVID-19, which has created a substantial burden on the afflicted HCWs themselves and on their families and society as a whole 5. Therefore, the early detection of infections in HCWs is of vital clinical significance.

Nevertheless, to our knowledge, studies investigating the clinical features of HCWs with COVID-19 are limited, with research focused mainly on personal-protection and mental health issues 6-10. Imaging for COVID-19 has been investigated in some radiological studies, although most have focused solely on the initial computed tomography (CT) signs 11-15, with dynamic changes over the course of disease largely unstudied. Four recent reports on the dynamic and evolving patterns in the imaging of COVID-19 aroused our attention 16-19. However, in these longitudinal studies, the chest CT features in patients at each stage of COVID-19 disease have not been investigated in detail. Furthermore, the sample sizes of some previous studies were relatively small, and the observed CT patterns (almost all progression-and-absorption) were single and biased 16, 17, 19. Hence, a longitudinal study with a large sample size was needed to obtain a comprehensive understanding of the clinical characteristics and the associated dynamic patterns of chest CT imaging features in HCWs diagnosed with COVID-19.

Here, a retrospective study of a cohort of HCWs with COVID-19 was performed. The clinical data and corresponding dynamic CT findings were investigated in detail to describe the clinical and imaging profiles of COVID-19 pneumonia disease progression. The aim of this study was to enable recognition of these cases in the each stage of disease.

## Methods

### Study design and participants

All the 40 medical workers were diagnosed in other hospitals and transferred to Wuhan Third Hospital (Tongren Hospital of Wuhan University), which was a designated hospital. All included patients were admitted from January 9, 2020, to February 10, 2020. This study was approved by the Research Ethics Commission of Wuhan Third Hospital, and patient informed consent was waived in accordance with the Council for International Organizations of Medical Sciences (CIOMS) guidelines.

### Laboratory procedures and CT image acquisition

Methods for identifying SARS-CoV-2 infection have been described previously 20. In brief, throat swab specimens were obtained from all patients at admission and tested using real-time reverse transcriptase-polymerase chain reaction (RT-PCR). The standards for discharge were absence of fever for more than 3 days, significant improvement in respiratory symptoms, obvious absorption in lesions in both lungs on CT images, and negative SARS-CoV-2 RNA status in two consecutive (at least 1 day apart) nucleic acid tests of throat swabs 21. Patients underwent routine blood tests, coagulation tests, serum biochemical tests (including renal, liver, and cardiac function), and tests for procalcitonin levels. The unenhanced chest single inspiratory phase CT images were acquired from patients in a supine position using either of two CT scanners (SOMATOM Definition AS, Siemens Healthineers, Erlangen, Germany; uCT 760, United Imaging, Shanghai, China).The CT protocols of SOMATOM Definition AS was as follows: 120 kVp, 200 mAs; slice thickness 5 mm; pitch, 0.625; matrix, 512×512, and the reconstruction section thickness was 0.6 mm. The CT protocols of uCT 760 were as follows: 120 kVp, 210 mAs; slice thickness 5 mm; pitch, 0.625; matrix, 512×512. The reconstruction section thickness was 0.5 mm, and the CT scans were collected from time of admission to discharge.

### Clinical data and CT imaging assessment

**Figure 1:**
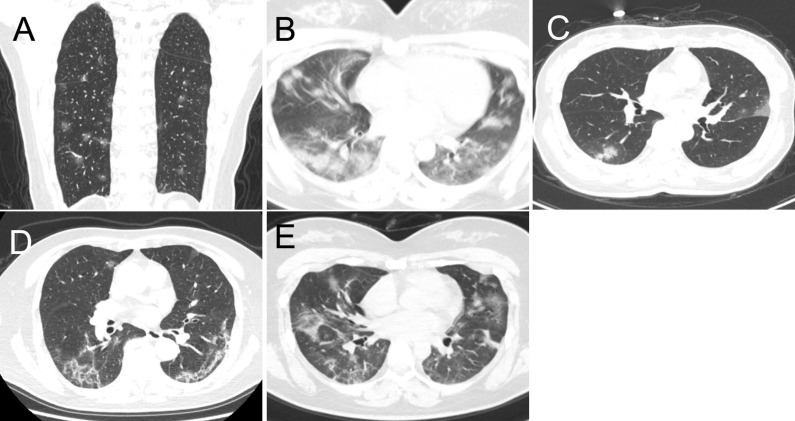
The predominant CT patterns of mild COVID-19 pneumonia. **A.** GGO. **B.** Consolidation. **C.** GGO and consolidation. **D.** Linear opacity. **E.** Linear opacity, GGO and consolidation.

Demographic data, epidemic-related factors, symptoms, underlying medical conditions, laboratory tests, and date of admission and discharge were collected from electronic medical records. If we encounter uncertain patients, we have confirmed the information via telephone or directly to communicate with each other. The CT images were evaluated by two radiologists with 15 and 10 years of experience in chest imaging; disagreements were resolved by consensus. All CT imaging features were defined according to the Fleischner Society Glossary of Terms in Thoracic Imaging as well as peer-reviewed literature on viral pneumonia 17, 22-24. The following imaging features were reviewed for all patients (the definition of CT was attached as a supplementary file ): lung involvement, extent of lesion involvement, predominant location, margin definition, lung segments of lesion distribution, number of lung segments and lobes involved, predominant CT pattern (Figure 1), and the presence of pure ground glass opacity (GGO), pure consolidation, GGO with consolidation, linear opacity, crazy-paving pattern, air bronchogram, reversed halo sign, nodules, thickening of the adjacent pleura, pleural effusion, thoracic lymphadenopathy, emphysema, round cystic changes, calcification, cavitation, bronchiectasis, and honeycomb pattern. A pre-established severity scoring system was applied 24. Each lung was divided into upper, lower, and middle zones demarcated as above the carina, below the inferior pulmonary vein, and between these, respectively (total of six lung zones). Scores for each zone were defined as follows: score 0, 0% involvement; score 1, less than 25% involvement; score 2, 25% to less than 50% involvement; score 3, 50% to less than 75% involvement; and score 4, 75% or greater involvement. Total scores were the sums of the six zones (range of possible scores, 0-24). In addition to total lesions score, we evaluated the individual total scores for GGO, consolidation, and linear opacity in all lesions. To obtain detailed information about the dynamic evolution of CT features with disease progression, we divided the time axis into four stages according to the interquartile range of the time from the onset of illness: stage 1 (1-6 days), stage 2 (7-13 days), stage 3 (14-22 days) and stage 4 (>22 days).

### Statistical Methods

All statistical analyses were performed using IBM SPSS Statistics Software (version 25; IBM, New York, USA). The clinical data are presented as the mean and standard deviation (SD) with a normal distribution, and median and interquartile range (IQR) with a non-normal distribution or distribution of the frequency with qualitative data. Categorical date was presented as frequency rates and percentages. Staging CT scores on different days were obtained through curve fitting module and interquartile range 16. *P* values < 0.05 with a two-tailed test were considered as statistically significant.

## Results

### Clinical features

According to the National Health Commission of the People's Republic of China Diagnosis and Treatment guidelines for COVID-19 21, 37 of the 40 HCWs were diagnosed as mild or common type, and three (8%) patients had converted to the severe type. All patients eventually met discharge criteria, and no deaths were documented. Of the 40 HCW cases, the median age was 40 years (IQR: 30, 47; Table 1), and 33 (83%) cases were female. More than half of the HCW cases were nurses (22, 55%). All cases were nosocomial infections acquired through close contact with COVID-19 patients. Only six of the infected HCWs had underlying medical comorbidities. The most common symptoms were fever (31, 78%) and cough (23, 58%), and the median hospital stay was 18 days (IQR: 13, 23). Abnormal laboratory tests included leukopenia (n=7, 18%), lymphocytopenia (14, 35%), increased alanine aminotransferase (9, 23%), aspartate aminotransferase (5, 13%), lactate dehydrogenase (5, 13%), C-reactive protein (19, 48%) and D-dimer (7, 27%; Table 2).

### Image findings

**Figure 2:**
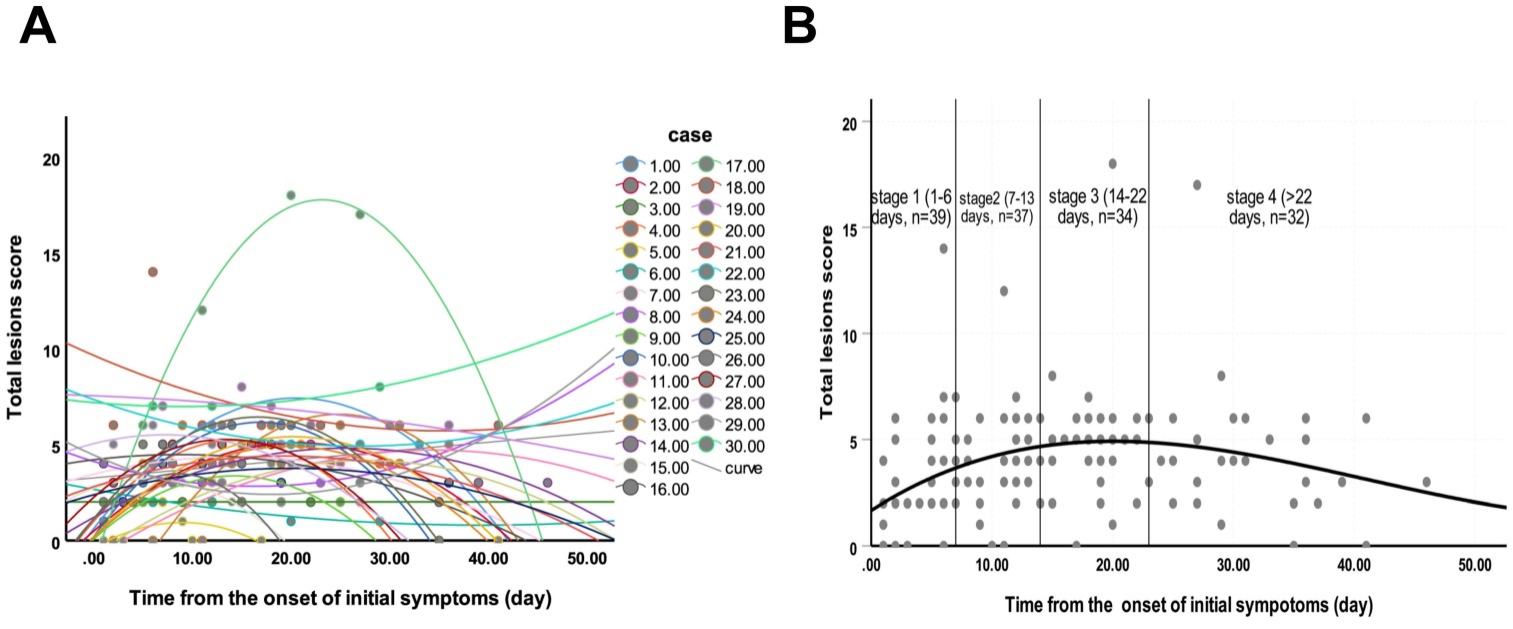
Changes of total lesions score from time of onset of initial symptoms (in days). **A.** The dynamic changes in total lesions score for each patient. **B.** Peak total lesions score appeared on day 20 (curve fitting equation: *y* = 1.667+0.364*x*-0.012*x*^2^+0.000098*x*^3^, in which x = time from onset of initial symptoms, y = total lesions score; *R^2^* = 0.12, *p*=0.001). Quartiles of patients between 0 and 46 days are shown as stages 1 to 4.

**Figure 3:**
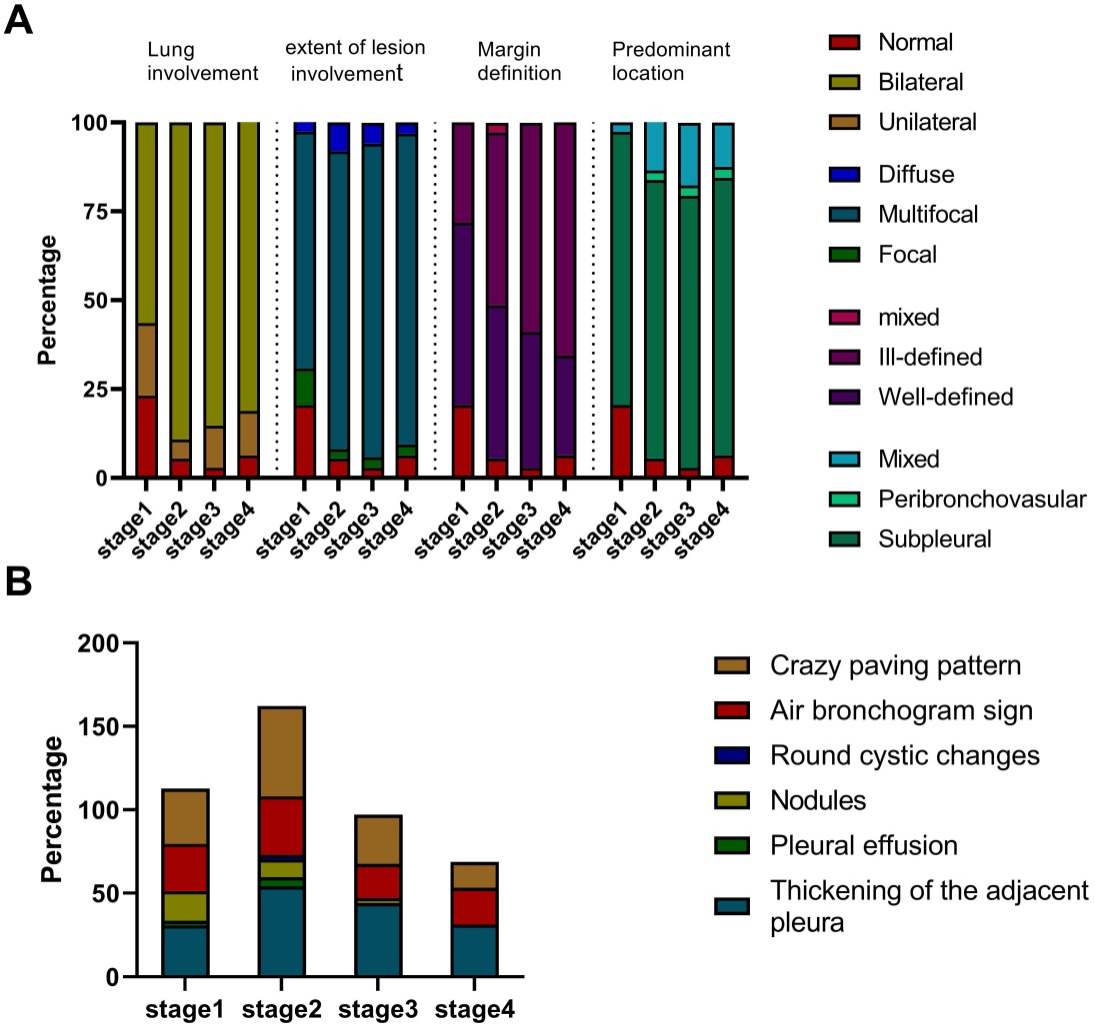
Imaging manifestation of 30 patients in each stage.** A.** The lung involvement, extent of lesion involvement, margin definition and predominant location of the lesions in each stage. **B.** The percentage of crazy-paving pattern, air bronchogram sign, round cystic changes, nodules, pleural effusion, and thickening pleura in each stage.

**Figure 4:**
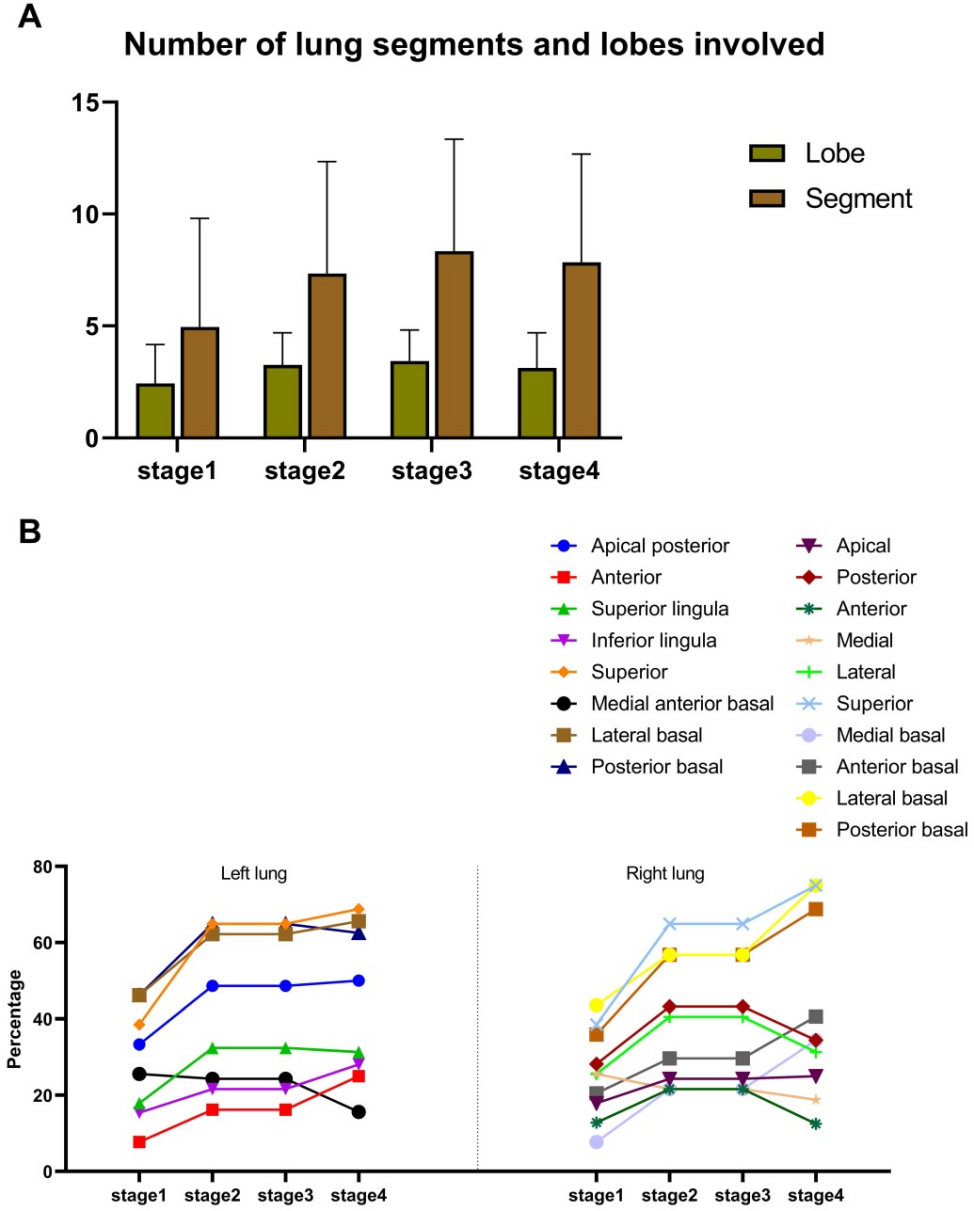
Distribution of lesions. **A.** Numbers of lung segment and lobes involved. **B.** Proportion of involvement in each lung segment.

**Figure 5:**
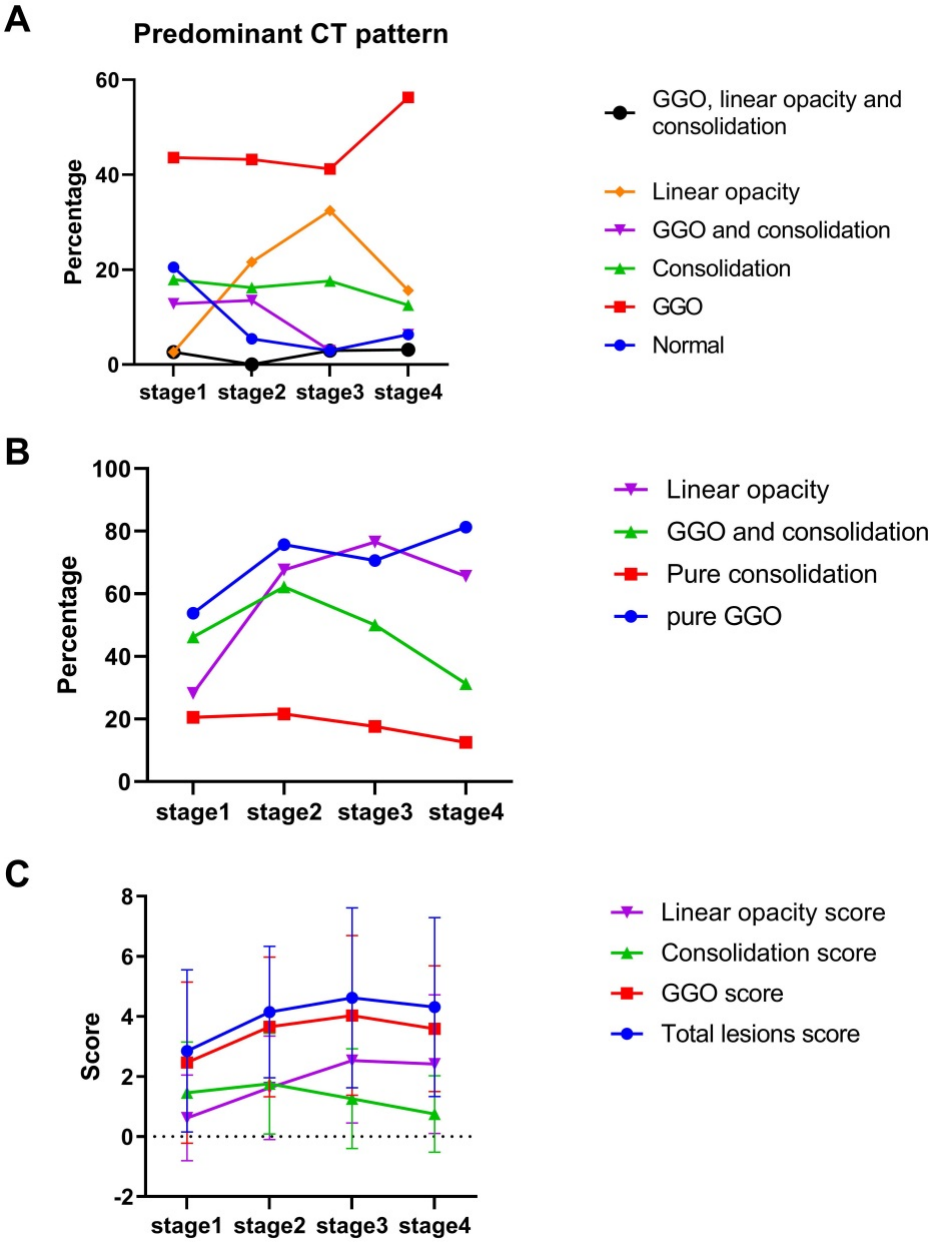
Quantitative evaluation of predominant computed tomography pattern. **A.** Percentage of predominant pattern in each stage. **B.** Percentage of pure ground glass opacity (GGO), pure consolidation, GGO and consolidation and linear opacity. **C.** Total lesions score, GGO, consolidation and linear opacity in each stage.

Of 40 HCWs with confirmed COVID-19, seven had negative CT images, and except for the 3 patients who became severe type, we finally enrolled 30 (75%) patients with 142 CT scans for images evaluation. Each stage included 39, 37, 34 and 32 CTs, respectively. The average total lesions score reached the highest number on the 20th day from the onset of illness (Figure 2). Three dynamic patterns were observed: progression-and-absorption (22, 73%), absorption-and-progression (6, 20%), and continuous absorption (2, 7%). The imaging features were divided into stages 1-4 (defined in Methods) based on time after onset of disease (Figures 3-5). Bilateral lesions were commonly observed in patients at all stages [22(56%) for stage 1, 33 (89%) for stage 2, 29 (85%) for stage 3 and 26 (81%) for stage 4], and unilateral lesions were commonly observed in stage 1 (8, 21%); Figure 3A). The lesions were primarily multifocal in each stage (26 (67%) for stage 1, 31 (84%) for stage 2, 30 (88%) for stage 3 and 28 (88%) for stage 4), while focal lesions appeared only in stage 1 (4, 10%). Over time, the well-defined lesions decreased from stage 1 to 4 [20 (51%) for stage 1, n=16 (43%) for stage 2, 13 (38%) for stage 3 and 9 (28%) for stage 4], while the ill-defined lesions increased (11 (28%) for stage1, 18 (49%) for stage 2, 20 (59%) for stage 3 and 21 (66%) for stage 4). Most lesions appeared in the subpleural areas at each stage (30 (77%) for stage 1, 29 (78%) for stage 2, 26 (76%) for stage 3 and 25 (78%) for stage 4). Pleural thickening, crazy-paving pattern, and air bronchograms occurred in all stages, and the patterns of change were consistent over time with the most common observations in stage 2 (n=20 (54%), 20 (54%) and 13 (35%), respectively; Figure 3B). Nodules were most commonly observed in stage 1 (7, 18%), then gradually decreased, and disappeared in stage 4. Pleural effusion was only observed in stages 1 (1, 3%) and 2 (2, 5%). Round cystic changes were only observed in stage 2 (1, 3%). Reversed halo sign, thoracic lymphadenopathy, emphysema, calcification, cavitation, bronchiectasis, and honeycomb pattern were not seen in this case series.

Moreover, the number of involved lung segments and lobes increased initially, reaching the maximum in stage 3 (mean value: 3 and 8, respectively) and then decreased, (Figure 4A). The superior segment was the most commonly involved site in both lungs (right lung: 15 (39%) for stage 1, 24 (65%) for stage 2, 25 (74%) for stage 3 and 24 (75%) for stage 4; left lung: 15 (39%) for stage 1, 24 (65%) for stage 2, 27 (79%) for stage 3 and 22 (69%) for stage 4), followed by the posterior basal segment (right lung: 14 (36%) for stage 1, 21 (57%) for stage 2, 23 (68%) for stage 3 and 22 (69%) for stage 4; left lung: 18 (46%) for stage 1, 24 (65%) for stage 2, 21 (62%) for stage 3 and 20 (63%) for stage 4) and the lateral basal segment (right lung: 17 (44%) for stage 1, 21 (57%) for stage 2, 26 (77%) for stage 3 and 24 (75%) for stage 4; left lung: 18 (46%) for stage 1, 23 (62%) for stage 2, 20 (59%) for stage 3 and 21 (66%) for stage 4) of lower lobes (Figure 4B).

The decrease-and-increase GGO pattern was observed in all stages (17 (44%) for stage 1, 16 (43%) for stage 2, 14 (41%) for stage 3 and 18 (56%) for stage 4; Figure 5A). The linear opacity increase-and-decrease pattern was observed in stage 1 (1, 3%), stage 2 (8, 22%), stage 3 (11, 32%) and stage 4 (5, 16%). The consolidation pattern was observed in stage 1 (7, 18%) and stage 3 (6, 18%). GGO with consolidation mixed pattern was observed in stage 1 (5, 13%) and stage 2 (5, 14%). Pure GGO described the highest proportion of lesions in each stage (21 (54%) for stage 1, 28 (76%) for stage 2, 24 (71%) for stage 3 and 26 (81%) for stage 4; Figure 5B). The consolidation score peaked in stage 2 (mean value: 1.8), and the total lesions scores peaked in stage 3 (mean value: 4.6; Figure 5C).

## Discussion

To the best of our knowledge, this is the first study to assess both clinical characteristics and longitudinal chest CT features of HCWs diagnosed with COVID-19, and distinct clinical characteristics and imaging features were observed in these patients 16, 17, 20. First, COVID-19-related pneumonia in HCWs was most common in young female workers (predominantly nurses) without underlying medical conditions, which indicates that, despite good physical health, these HCWs were at an increased risk of infection. Second, the total lesions score peaked on the 20th day after the onset of symptoms, a time interval longer than had been previously documented. Third, the CT lung consolidation scores were a better reflection of the disease severity than were the total lesion scores 16, 18, 19, which suggests that a reasonable scoring system should be based on a comprehensive assessment of the lesion both in its extent and its pathological nature.

Our finding of a predilection for the younger female HCWs without underlying medical conditions was inconsistent with previous studies reporting that older male patients with diabetes, cancer or severe cardiovascular diseases tend to be the most commonly affected by this disease 17, 20. This discrepancy might be due to differences in demographic data. In our study, more than half of the cases were nurses under 50 years old with no severe underlying medical conditions such as tuberculosis, cancer or chronic lung disease in their medical records. The HCWs in this study, especially the nurses, had been in close contact with COVID-19 patient airway and digestive tract secretions, which in turn increased the risk of infection with SARS-CoV-2 and subsequent development of COVID-19 pneumonia. Fever and cough were the most common symptoms which are similar to clinical symptoms observed in other infectious diseases affecting the lower respiratory tract 19, 25. Nevertheless, upper respiratory tract symptoms (such as rhinorrhea and sore throat) and non-specific symptoms (such as fatigue, diarrhea, and headache) were observed in 10%-45% of cases. Therefore, symptoms alone are insufficient to diagnose an individual with COVID-19. Previous studies not involving HCWs revealed alterations in the laboratory index (including leukopenia, lymphocytopenia, increased alanine aminotransferase, increased aspartate aminotransferase, increased lactate dehydrogenase, and increased C-reactive protein and D-dimer) similar to those observed in our study 20, 26. Additionally, the length of stay in the hospital was shorter in our cohort than that reported for a previous non-HCW cohort, which may be partially because there were fewer severe cases in our study 26.

In the present study, 142 CT scans of 30 HCW COVID-19 patients were divided into four stages based on time from the onset of symptoms. The total lesions score peaked on the 20th day (stage 3), with the time interval longer than the 6-11 days documented in previous studies 16, 18. The reasons for this phenomenon could be summarized as follows: First, unlike non-HCW cases, the HCWs, along with their medical knowledge, had access to timely detection and intervention for COVID-19 pneumonia. This could explain the prolonged interval from illness onset to the highest total lesions score observed in this study. Second, the largest number of involved pulmonary segments at stage 3 in this study provided direct evidence that an area-based total lesions score could peak on the 20^th^ day. Third, reports of a time interval of 12 (8-14) days from onset of illness to severe clinical pneumonia together with those of the 1- or 2-week delay in alterations on CT following the clinical transition to severe pneumonia 19, 26 provide further evidence for the delayed time to peak of the total lesions score in the present study. In a previous study, the median duration of viral shedding after COVID-19 onset was nearly 20 days, which indicates that the disease would enter the severe stage during this period 26. We speculate that the alterations in viral quantity and virulence may be the pathological basis underlying the evolving pattern of CT total lesions score with disease progression. However, future studies should clarify the link between these factors and CT performance 17, 19.

Our findings of multifocal, subpleural areas in lower lobes with a prominent GGO pattern were suggestive of COVID-19 pneumonia and consistent with findings in previous studies 14, 16, 17. However, the dynamic CT features with COVID-19 pneumonia progression has been largely uninvestigated. In this study, several interesting dynamic CT findings were observed. First, a gradual ill-defined tendency in the boundaries of lesions from stage 1 and stage 2 to stage 3 and stage 4 indicated that more lesions were absorbed with time after onset of illness. Second, the CT features at the early stage (stage 1) of COVID-19 pneumonia were nonspecific (such as nodules). Conversely, in the advanced stages of COVID-19 pneumonia, specific changes in the imaging pattern could be detected (for example, the GGO gradually solidified and the CT signs of crazy-paving pattern and reticular linear opacity were more obvious) 27. Therefore, a CT imaging-based exclusion of COVID-19 pneumonia should be treated with caution, especially in the earlier stages of disease. Third, we revealed that the consolidation score, rather than total lesions score, could objectively reflect the severity of lung involvement. Reportedly, GGO on CT may represent early alveolar damage with exudation, whereas consolidation is the manifestation of severe lung involvement 28. As such, for the mild and common subtype cases, the consolidation score may be a better indicator of the severity of lung involvement than the total lesions score. Furthermore, CT signs reflecting the severity of lung involvement (such as crazy-paving pattern, pure consolidation and air bronchogram) all peaked in stage 2, as did the consolidation score. However, the total lesions score did not peak until stage 3. The exact cause of this inconsistency remains unclear. One possible reason is that the area of total lesions may be enlarged in the transition from consolidation to GGO; future investigations will be needed to investigate this issue further. Taken together, these findings indicate that the stage 2 of COVID-19 pneumonia may be the critical period to control the disease because the largest involved lung area occurred in stage 3.

In conclusion, COVID-19 pneumonia in HCWs at Wuhan Third Hospital most commonly affected younger female workers. In these patients, stage 2 (7-13 days after onset) of COVID-19 pneumonia may be the most critical period for controlling the progression to severe disease, and the consolidation scores could objectively reflect the severity of the lung involvement and thus serve as a more accurate diagnostic measure than total lesions scores at each stage.

## Supplementary Material

Supplementary figures and tables.

## Figures and Tables

**Table 1:** Clinical characteristics of 40 healthcare workers who were patients

Demographics	
Age, years	40 (30, 47)
**Sex**	
Female	33 (83%)
Male	7 (18%)
**Clinical features**	
***Symptoms***	
Fever	31 (78%)
Fatigue	18 (45%)
Short of breath	8 (20%)
Headache	5 (13%)
Inappetence	4 (10%)
Chills	3 (8%)
Rhinorrhea	3 (8%)
Nausea	2 (5%)
Cough	23 (58%)
Expectoration	12 (30%)
Joint soreness	7 (18%)
Diarrhea	4 (10%)
Muscle soreness	4 (10%)
Sore throat	3 (8%)
Palpitation	1 (3%)
***Comorbidity***	
Hypertension	3 (8%)
PTB	1 (3%)
Hypohepatia	1 (3%)
CHD	1 (3%)
SLE	1 (3%)
***Inpatient days***	18 (13, 23)

Data are median (IQR), n (%). CHD: coronary heart disease; PTB: pulmonary tuberculosis; SLE: systemic lupus erythematosus.

**Table 2:** Laboratory findings of 40 healthcare workers who were patients

Laboratory examinations	N	Reference range	Median (interquartile range)	Increased	Decreased
White blood cell count, × 10⁹ per L	40	3.5-9.5	4.7 (3.7, 6.8)	1 (3%)	7 (18%)
Lymphocyte count,× 10⁹ per L	40	1.1-3.2	1.3 (1, 1.8)	1 (3%)	14 (35%)
Platelet count, × 10⁹ per L	40	125-350	228 (180.9, 273.8)	2 (5%)	1 (3%)
C-reactive protein, mg/L	40	0-5	4.7 (0.8, 12.7)	19 (48%)	
Alanine aminotransferase, IU/L	40	9-50	20.5 (13, 32)	9 (23%)	
Aspartate aminotransferase, IU/L	40	0-45	21 (16.25, 33)	5 (13%)	
Lactate dehydrogenase, IU/L	40	114-240	173.5 (150.8, 193)	5 (13%)	1 (3%)
Total bilirubin, µmol/L	40	2-21	7.2 (5.5, 10.2)		
Creatinine, µmol/L	40	40-105	53.8 (48.1, 63.1)		2 (5%)
Creatine kinase, IU/L	40	30-180	63 (43.5, 87)	1 (3%)	
PT, s	24	10-13	11.3 (10.9 11.7)		
APTT, s	24	21-35	27.5 (26.3, 31.9)		
D-dimer, μg/mL	26	0-0.5	0.3 (0.2, 0.7)	7 (27%)	
Procalcitonin, ng/mL	35	<0.05	<0.05		

Data are median (IQR), n (%). PT: Prothrombin time; APTT: Activated partial thromboplastin time.

## Abbreviations

COVID-19: Coronavirus Disease 2019
HCWs: healthcare workers
GGO: ground glass opacity
IQR: interquartile range
