# Supplementary material for: Clinical characteristics and longitudinal chest CT features of healthcare workers hospitalized with coronavirus disease 2019 (COVID-19)
Source: Int J Med Sci. 2020 Sep 21;17(17):2644–52. doi: 10.7150/ijms.48696 (PMC7645347; doi:10.7150/ijms.48696)
Supplement: Supplementary file 1 — Supplementary figures and tables. [file ijmsv17p2644s1.pdf]

S1:

CT characteristics	Definition
Lung involvement	Categorized as bilateral or unilateral lung involvement
Extent of lesion involvement	Categorized as diffuse, multifocal or focal
Predominant location	Categorized as subpleural, peribronchovascular or mixed
Margin definition	Categorized as well-defined or ill-defined
Predominant CT patternair bronchogram	Ground glass opacity pattern, it appears as hazy increased opacity of lung, with preservation of bronchial and vascular margins; Consolidation pattern, appears as a homogeneous increase in pulmonary parenchymal attenuation that obscures the margins of vessels and airway walls; Ground glass opacity and consolidation pattern, combination of ground glass opacity and consolidation; Linear opacity pattern, interstitial lesions of pulmonary parenchyma, and can be interwoven into a reticular opacity; Ground glass opacity,consolidation pattern and linear opacity pattern, combination of consolidation, ground glass opacities, and linear opacities
Crazy-paving pattern	This pattern appears as thickened interlobular septa and intralobular lines superimposed on a background of ground-glass opacity
Air bronchogram	A pattern of air-filled (low-attenuation) bronchi on a background of opaque (high-attenuation) airless lung
Reversed halo sign	A focal rounded area of ground-glass opacity surrounded by a more or less complete ring of consolidation
Thoracic lymphadenopathy	Thoracic lymphadenopathy (hilar or mediastinal) with short-axis diameter greater than 1 cm
Round cystic changes	a small gas-containing space within lesion
Cavitation	A cavity is a gas-filled space, seen as a lucency or low-attenuation area, within lesion
Bronchiectasis	Bronchiolectasis is defined as dilatation of bronchioles
Honeycomb pattern	Honeycombing represents destroyed and fibrotic lung tissue containing numerous cystic airspaces with thick fibrous walls
